# Possibilities and Limitations of ICP-Spectrometric Determination of the Total Content of Tin and Its Inorganic and Organic Speciations in Waters with Different Salinity Levels—Part 2: Separate Determination of Inorganic and Organic Speciations of Tin

**DOI:** 10.3390/molecules28186615

**Published:** 2023-09-14

**Authors:** Zaual Temerdashev, Pavel Abakumov, Mikhail Bolshov, Darya Abakumova, Alexander Pupyshev

**Affiliations:** 1 Analytical Chemistry Department, Faculty of Chemistry and High Technologies, Kuban State University, 350040 Krasnodar, Russia; pg.abakumov@gmail.com (P.A.); abakumova.dd@gmail.com (D.A.); 2 Institute for Spectroscopy Russian Academy of Sciences, 108840 Moscow, Russia; mbolshov@mail.ru; 3 Department of Physical and Chemical Methods of Analysis, Institute of Physics and Technology, Ural Federal University, 620062 Yekaterinburg, Russia; pupyshev@gmail.com

**Keywords:** inorganic speciation of tin, organotin compounds, hydride generation of tin, sorption, solid-phase separation

## Abstract

In this study, determination of the inorganic and organic forms of tin in waters of different salinities is considered. The possibility of the separation of speciations of tin using liquid–liquid extraction (LLE); precipitation with fluorides, iodides, ammonia, and iron (III) chloride; and sorption of organotin compounds (OTCs) was studied. The LLE and analyte precipitation methods proved to be ineffective. Inorganic and organic forms of tin were separated by the sorption of OTCs using silica gel sorbent Diapak C18. Under optimized conditions, a technique for the separate determination of the forms of tin in natural waters was developed. The technique combines hydride generation and microwave mineralization of solutions followed by ICP spectrometry. The inorganic forms of tin were determined after their solid-phase separation from organotin compounds. The lower limits of analyte quantification were 0.03 μg/L (ICP-MS) and 0.05 μg/L (ICP-OES), which provide separate determinations of inorganic and organic forms of tin in waters with different salinities. The content of OTCs in water was determined by subtracting the inorganic concentration from the total concentration of tin. The technique will allow a comprehensive assessment of the toxicological impact of tin speciations on the aquatic ecosystem.

## 1. Introduction

The natural tin supply in the environment comes from metal-containing minerals and anthropogenic sources [1]. The toxicity and biological activity of tin are related to the degree of oxidation of the inorganic forms and the degree of methylation of the organic compounds; therefore, the assessment of environmental pollution requires the determination of tin speciations [1,2]. Because the toxic impact of various forms of tin on living organisms is different, it is necessary to determine the speciations of the analyte to adequately estimate the levels of water pollution [2,3,4].

In fresh- and seawater, inorganic tin is predominantly present in the tetravalent form [5]. Inorganic tin compounds exhibit a toxic effect at relatively high concentrations [3]. On the other hand, as a result of chemical and biochemical transformations in the environment, they can transform into toxic organotin compounds (OTCs), leading to adverse effects on marine and freshwater organisms [5,6]. OTCs are present in water in the form of covalently bonded alkyl or aryl groups of tetravalent tin: methyl, ethyl, butyl, propyl, and phenyl. Their molecular formulas have the form R_4_Sn, R_3_SnX, R_2_SnX_2_, and RSnX_3_, where R is a functional group and X is a counterion, for example, halide, oxide, or hydroxide [7,8,9]. In the environment, OTCs are thermally stable because the C-Sn bond is stable up to 200 °C and the number of Sn-C bonds and the length of the alkyl chains strongly affect the chemical and physical properties of OTCs [2,10,11].

Many countries control environmental pollution in natural waters [12], and the International Maritime Organization [13] introduced a ban on the use of antifouling paints based on tributyltin on all marine vessels in 2008 (Table 1).

Table 1 summarizes the legislative regulations in different countries on the hourly and daily average of tributyl alcohol concentrations in various types of aquatic ecosystems. We have not found limitations for other inorganic and organic analytes. The national legislation of Russia has established restrictions on inorganic and organic content for fishery reservoirs, which amount to 112 μg/L for inorganic tin and from 0.01 to 10 μg/L for organotin compounds [19].

Spectrometric methods play an important role in the determination of tin in the aquatic environment due to their low detection limits. However, the matrix of seawater significantly reduces the analytical signal of the analyte, which requires additional stages of sample preparation. ICP-MS and ICP-OES are highly sensitive methods of analysis, but they provide information on the total contents of all forms of the analyte [20]. The determination of low concentrations of tin in highly saline waters using ICP-OES and ICP-MS methods is difficult because of matrix and spectral interference, a decrease in the ionization efficiency, and clogging of the atomizer and burner injector. ICP-spectrometric determination of organic and inorganic speciations of tin in water is possible only by introducing a preliminary separation stage. For example, ICP spectrometry is used in the determination of OTCs in combination with gas chromatography [21,22,23] or gas chromatography with mass spectrometry [24,25,26] after analyte derivatization.

Liquid–liquid (LLE) and solid-phase (SPE) extraction techniques are used to extract the OTCs from the aqueous matrix. When assessing the total content of organic and inorganic speciations, it is also necessary to consider the possible recovery of inorganic tin into the organic phase [4,7,12,27]. One of the methods for separating tin speciations can be the precipitation of OTCs with halides from an aqueous medium [10,28,29,30]. However, the possibility of separating various speciations of tin under conditions of low analyte concentrations has not been sufficiently studied. We also note that the determination of organic and inorganic forms of tin requires different schemes for preparing seawater for analysis.

In the first part of our research, detailed information on the methods for determining tin forms, the limits of quantification, and the results of the analysis of waters of the Azov and Black Seas are presented. The possibilities and limitations of the ICP-spectrometric determination of the total tin content and its inorganic and organic forms in waters with different levels of salinity are considered [31]. The total tin content in water samples of the Azov (0.17 μg/L) and Black (0.24 μg/L) Seas was measured using the developed analytical schemes. In this paper, we discuss the possibility of determining the inorganic and organic speciations of tin in water using a combination of ICP spectrometry with different schemes of sample pretreatment. Methods for separating and preconcentrating, reducing, and/or eliminating matrix effects in the determination of an analyte using ICP-OES and ICP-MS, as well as methods for determining the total tin content in seawaters of various salinities, are considered.

The results of these studies will provide an analytical basis for a comprehensive assessment of the toxicological effects of tin on the aquatic ecosystem under study.

## 2. Results and Discussion

The separate determination of inorganic and organic forms of tin is an important task in the ecoanalytical monitoring of aquatic systems because different forms of tin have different toxicities. The use of ICP spectrometry for the separate determination of organic and inorganic forms of tin in seawaters is possible only after solving the problem of their preliminary separation. In this part of the study, possible approaches for the separation of organic and inorganic tin in the matrix of surface waters are investigated.

### 2.1. Assessment of the Possibility of Tin Forms Separation by Precipitation from Aqueous Media

The possibility of isolating OTCs into slightly dissociated complexes was studied using solutions of analytes in deionized water containing 1.0 µg/L tin (IV) chloride; 0.25 µg/L TBT, TeBT, TMT, and MPT; and mixtures of OTCs with tin (IV) chloride, each 0.20 μg/L SnCl_4_, TBT, TeBT, TMT, and MPT. The analytes were precipitated with fluoride and potassium iodide at concentrations of precipitants from 1.0 μg/L to 4.0 g/L in terms of halide ions at room temperature and at 95 °C. The time of precipitation formation varied in the range of 1–30 min. In the entire range of the tested halide concentrations, no precipitation was observed in both cases within 30 min. However, when an excess of fluorides (0.5–4.0 g/L) was introduced into the samples, turbidity of the studied solutions was noticed, which did not disappear upon heating. It may indicate the formation of a colloidal phase of the OTC. However, after centrifugation of the resulting solutions at 1500 rpm, phase separation did not occur.

The replacement of the counterions of the OTC by F^−^ and I^−^ in the studied range of analyte concentrations also did not cause a change in the concentration of tin in the solution. This is evidenced by the experimental data, visual assessment of the precipitate in solutions, and the results of analysis using combined methods of ICP spectrometry with hydride generation after centrifugation and microwave digestion of the resulting solutions (Table 2).

Precipitation by halide binding probably occurs at higher OTC concentrations. This is indirectly confirmed by the values of the stability constants of the organofluorine complex compounds of tin in aqueous solutions, which on average are ~4.5 × 10^2^ [10,29]. The described experiments showed that the method of precipitation with the halides used was not effective for the separation of tin forms.

As an alternative separation option, the precipitation of tin (IV) chloride with aqueous solutions of ammonia and iron (III) chloride to form α-tin acid was considered [30]. The possibility of separating the precipitate was evaluated under similar conditions as in the case of precipitation of OTCs with halides. The formation of an insoluble tin compound was not visually observed. This was also confirmed by the results of ICP-MS and ICP-OES determination of tin with hydride generation after centrifugation of the solutions (Table 2).

Known precipitation methods failed to separate the inorganic and organic forms of tin in water. Apparently, this is due to the low concentrations of the formed compounds, at which their quantitative precipitation is difficult. In addition, under conditions of high salinity in seawater (chloride concentration of about 6–20 g/kg), a competing reaction of changing chloride counterions of the OTCs to fluoride or iodide is unlikely.

### 2.2. Liquid–Liquid Separation of Tin Forms in Water

**Figure 1:**
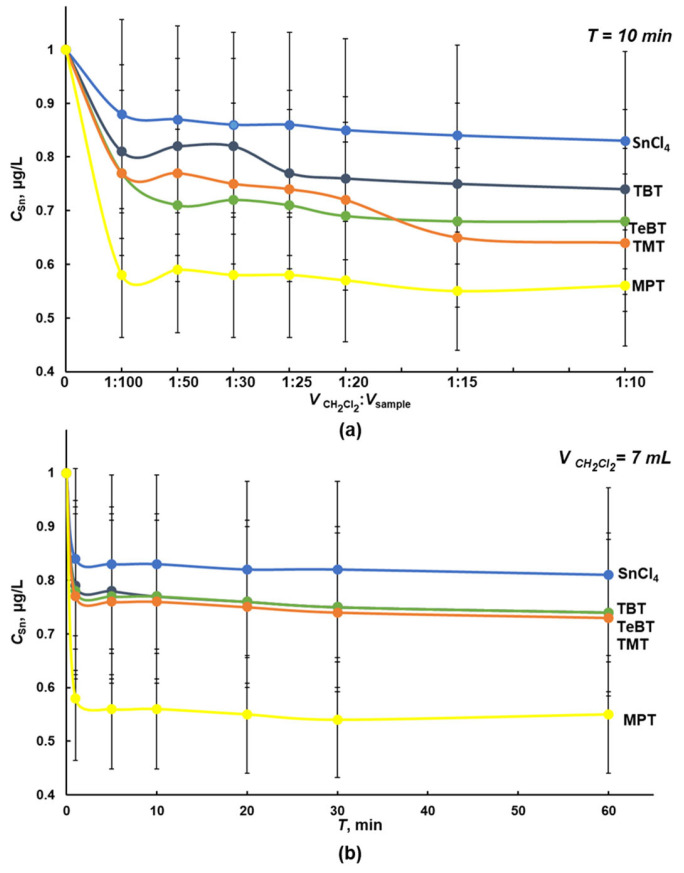
Influence of volume (**a**) and duration (**b**) of extraction with dichloromethane of tin (IV) chloride and OTCs from a model mixture of tin (IV) chloride, TBT, TeBT, TMT, and MPT, with each analyte concentration of 1.0 μg/L in terms of tin in deionized water (n = 3, *p* = 0.95).

**Figure 2:**
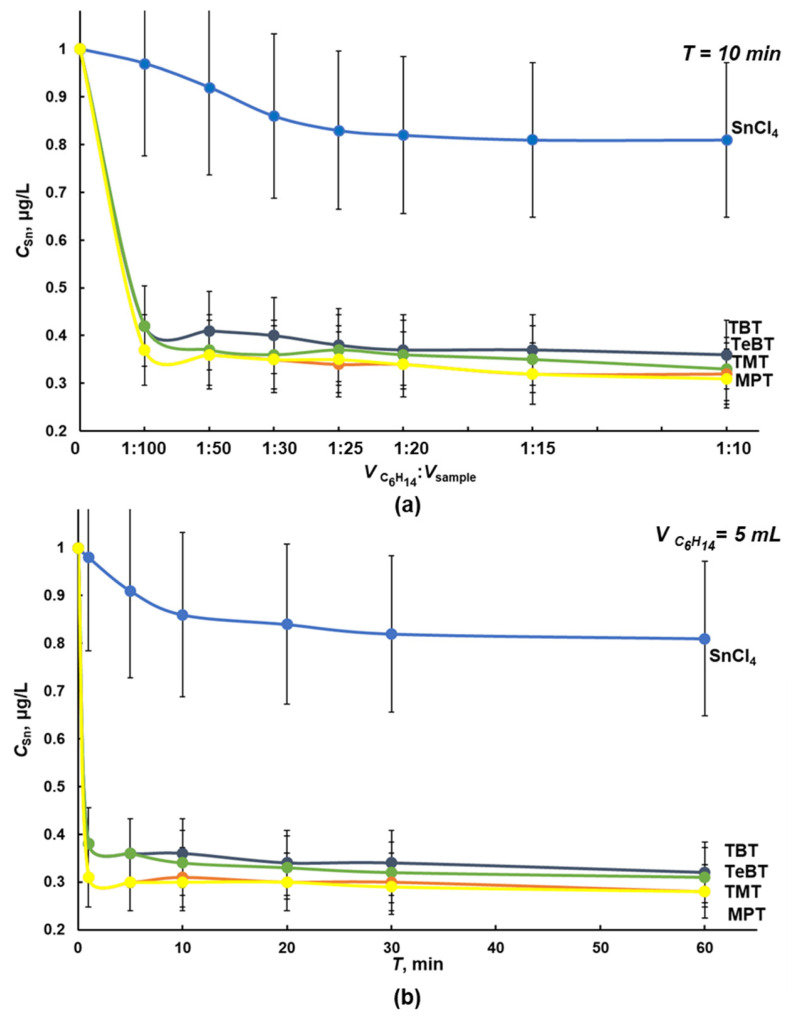
Influence of volume (**a**) and duration (**b**) of hexane extraction of tin (IV) chloride and OTCs from a model solution of deionized water with tin (IV) chloride, TBT, TeBT, TMT, and MPT, with each analyte concentration of 1.0 μg/L in terms of tin (n = 3, *p* = 0.95).

Dichloromethane and hexane provide efficient and rapid separation of organic and inorganic forms of tin during liquid–liquid extraction (LLE) of OTCs from water systems [13,22,23,25,32]. Separation of tin forms from solutions of 100 mL with 1.0 μg/L in terms of TBT, TeBT, TMT, MPT, and tin (IV) chloride in deionized water was studied depending on the extractant volume (Figure 1a and Figure 2a) and the extraction time (Figure 1b and Figure 2b). The concentration of tin in the aqueous phase after LLE and microwave digestion was controlled using ICP spectrometry with hydride generation.

The increase in the volume of extractants and the extraction time did not affect the efficiency of the extraction of OTCs from the solutions. Under optimal conditions (V(CH_2_Cl_2_) = 7 mL, T = 10 min), the dichloromethane extracted from solutions 25% of the total content of TBT, TeBT, and TMT, and about 45% of the total content of MPT (Figure 1). The low extraction efficiency is probably due to the relatively high solubility of the solvent in water (1.6% at 20 °C) [20]. We also noted that with an increase in the polarity of the solvent, the amount of co-extractable substances increases, which reduces the selectivity of the extraction of OTCs [32]. Apparently, this is the reason for the rather high degree of extraction of inorganic tin: 15–20% (Figure 1).

Hexane extracts up to 80% of each OTC due to the low solubility of the extractant in water (0.014% at 20 °C) and the lower polarity compared with dichloromethane [30,33] (Figure 2). The concentration of inorganic tin extracted with hexane is lower than with dichloromethane: 10–15%. Efficient LLE extraction of OTCs is achieved with 5.0 mL of hexane for 10 min during one cycle.

A possible increase in the degree of extraction of OTCs from aqueous solutions using dichloromethane and hexane was studied with the addition of sodium chloride [32]. The level of salinity of the studied solutions and the multiplicity of extraction did not affect the efficiency of OTC extraction.

It can be concluded that it is not possible to selectively isolate OTCs with LLE in the presence of inorganic tin compounds. In these cases, the authors [33] have proposed to introduce corrections for “hexane-extractable tin”, considering the co-extraction into the organic phase of the inorganic form of tin together with the OTC. Here, the possibility of analyzing real samples with unknown contents of tin forms and the algorithm of accounting for co-extraction becomes problematic. Hexane extracted no more than 80% (Figure 2) of OTCs from aqueous solutions due to non-polar interactions and the presence of counterions [2]. Therefore, LLE is often carried out in acidified solutions (acetic or hydrochloric acid) as well as with complexing agents (carbamates or tropolone), which allows an increase in the selectivity of the OTC extraction by changing the properties of the system [2]. Such approaches have their limitations; for example, complexing agents cannot be used at low pH, and they also increase the solubility of the co-extractable compounds [2,13].

### 2.3. Solid-Phase Separation of Tin Forms in Water

Solid-phase separation of tin compounds was tested on solutions of deionized water containing 1.0 μg/L tin (IV) chloride and mixtures of OTCs with a total tin analyte concentration of 1.0 μg/L (0.25 μg/L each of TBT, TeBT, TMT, and MPT). The pH values of the prepared solutions were in the range of 4–5. Initially, sorbents that did not adsorb inorganic tin, but retained OTCs, were chosen. After microwave digestion of all solutions, the analyte was determined using ICP spectrometry with hydride generation.

**Figure 3:**
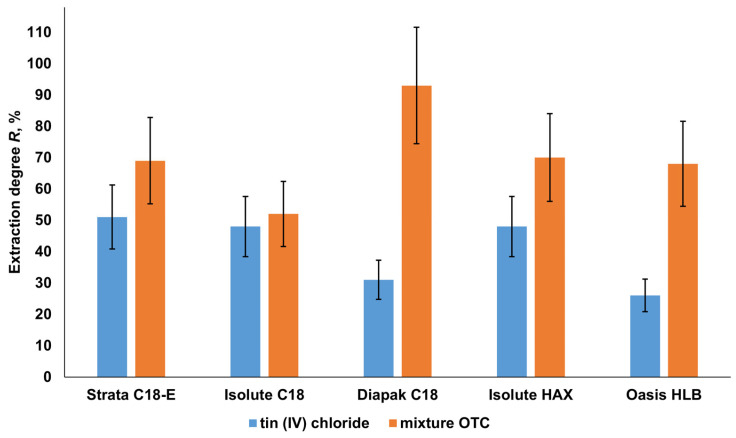
Degrees of extraction of OTCs and tin (IV) chloride by sorbents from solutions in deionized water (pH 4–5) with 1.0 µg/L tin (IV) chloride and 0.25 µg/L TBT, TeBT, TMT, and MPT (each) in terms of tin (n = 3, *p* = 0.95).

The sorbents Strata C 18-E, Isolute C 18, and Isolute HAX (C8) extracted from the model water samples at pH 4–5 from ~ 50 to 70% of OTCs at a degree of extraction of the inorganic forms of tin of 50% (Figure 3). The sorbents Diapak C18 and Oasis HLB provided the lowest retention of inorganic tin and the highest recovery of OTCs.

The mechanism of retention of inorganic tin by sorbents can be explained by their interaction with the analyte. Inorganic tin can be retained on sorbents that have functional groups in their structure, for example, silanol groups, which cause secondary anion exchange interactions with analytes. Mono-, di-, and trisubstituted OTCs at pH 4–5 can also be hydrolyzed and exist in the cationic form [34]. It can be assumed that the pH value of the analyzed solutions is of paramount importance in the solid-phase separation of the inorganic and organic forms of tin and determines the mechanism of their sorption [33,34].

The Diapak C18 sorbent extracted about 90% of the OTCs and up to 30% of the inorganic form of tin from the analyzed samples at pH 4–5 (Figure 3). The Oasis HLB cartridge, based on a polymer sorbent, extracted up to 25% of inorganic tin with an OTC extraction rate of 68%. Taking into account the possible influence of pH on the separation of tin forms, the change in the degree of extraction of the OTCs and the inorganic form of tin by these sorbents was investigated in a wide pH range. 

**Figure 4:**
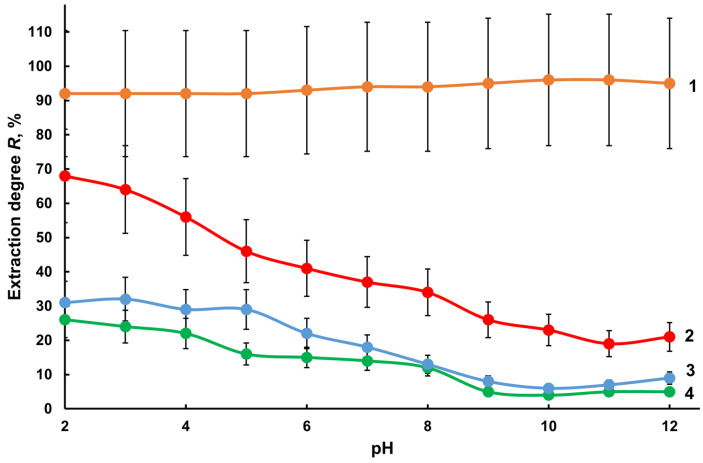
Influence of pH on the degree of extraction of OTCs by sorbents Diapak C18 (1) and Oasis HLB (2) and of tin (IV) chloride by sorbents Oasis HLB (3) and Diapak C18 (4) from solutions in deionized water with 1.0 μg/L tin (IV) chloride and 0.25 µg/L TBT, TeBT, TMT, and MPT (each) in terms of tin (n = 3, *p* = 0.95).

The sorption properties of the Diapak C18 and Oasis HLB cartridges were studied in the range from 2.0 to 12.0 pH units on model water samples containing 1.0 µg/L tin (IV) chloride, 0.25 µg/L TBT, TeBT, TMT, and MPT each with a total tin concentration of 1.0 µg/L (Figure 4). The pH of the solutions was regulated using hydrochloric acid and an aqueous solution of ammonia. The solutions were passed through the sorbents Diapak C 18 and Oasis HLB, the loaded aqueous phase was subjected to microwave mineralization, and the degree of tin extraction was determined using ICP-MS and ICP-OES with hydride generation. With the Diapak C18 sorbent in the pH range of 9–12, the degree of extraction of the OTCs was more than 95% and of inorganic tin less than 5% (Figure 4, curves 1 and 4). The shift to the alkaline region reduced the degree of extraction with the Oasis HLB sorbent not only of inorganic tin (Figure 4, curve 3), but also of OTCs (Figure 4, curve 2). Based on our experimental findings, this effect can mostly be explained not by the chemical forms involved in the sorption of analytes, but by the polymer base of the Oasis HLB, which has less selectivity to OTCs under the described conditions.

**Figure 5:**
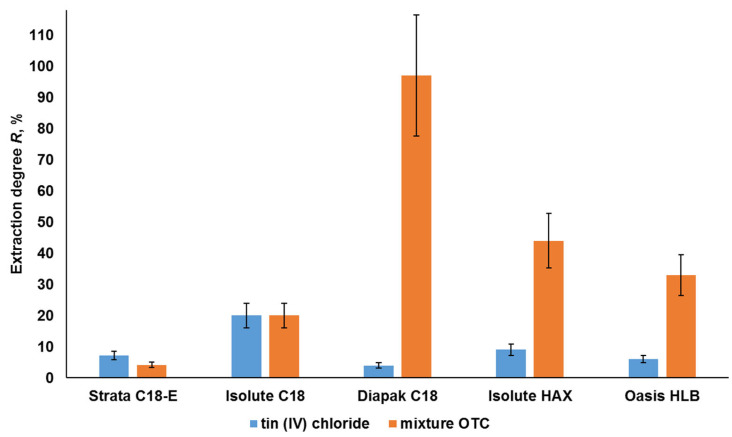
Degrees of extraction of tin forms by sorbents at pH = 10 from solutions in deionized water containing 0.20 µg/L tin (IV) chloride, TBT, TeBT, TMT, and MPT in terms of tin (n = 3, *p* = 0.95).

Considering the low degree of extraction of inorganic tin at alkaline pH (Figure 4), the possibility of separating analytes with the considered cartridges at pH = 10 from model solutions of deionized water containing 0.20 µg/L tin (IV) chloride, TBT, TeBT, TMT, and MPT was studied (Figure 5).

The efficiency of the cartridges based on silica gel sorbents for the extraction of tin forms, except for Diapak C18, is limited to the pH range of 2–9 [35]. At high pH values, the sorbents are destroyed and sorption properties change, which limits their use for separating organic and inorganic forms of tin. The sorbent Diapak C18 retained its properties at pH more than 9, which was proved by multiple successive measurements.

**Figure 6:**
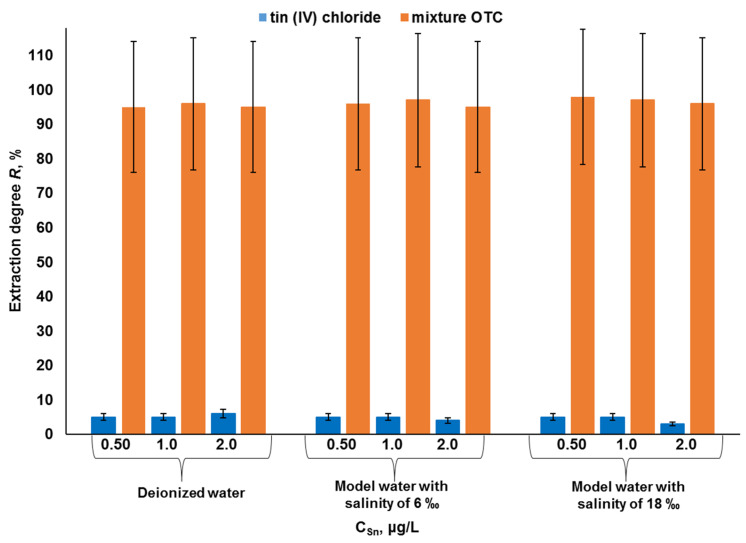
Degrees of extraction of inorganic and organic forms of tin in solutions based on deionized water and model marine water with salinities of 6 ‰ and 18 ‰ at pH = 10 (n = 3, *p* = 0.95).

The possibilities of selective solid-phase separation of tin forms on the Diapak C18 sorbent from the matrix of marine water were studied on model samples with different salinities at pH = 10, containing tin (IV) chloride as well as a mixture of OTCs (Figure 6). The amounts of tin (IV) chloride additives as well as OTC mixtures were 0.50, 1.0, and 2.0 μg/L in terms of tin.

The Diapak C18 sorbent selectively separated the chemical forms of tin both in deionized water and in model marine waters with salinities of 6 and 18 ‰ (Figure 6). For all analyzed model water samples, the degree of extraction of OTCs was more than 95% and that of inorganic tin was less than 5%, which confirms the selectivity of the separation of analyte forms in aqueous matrices with different salinities.

In real marine water, OTCs are less common than the inorganic form of tin, due to the course of biogeochemical processes accompanied by the degradation of OTCs, their absorption by biota, or their sedimentation [2,13]. Based on this, the possibility of selective solid-phase separation of tin forms on the Diapak C18 sorbent in solutions with various ratios of inorganic tin and OTCs (Table 3) was evaluated.

As can be seen, the ratios of the forms of tin of different natures in the analyzed samples do not affect the efficiency of their separation on the Diapak C18 sorbent, which is due to the fact that the inorganic tin is not retained by this sorbent and does not affect its sorption properties. In turn, the efficiency of OTC sorption on the Diapak C18 remains unchanged both at low (Table 3) and relatively high concentrations of analytes (Figure 6), which indicates a high sorption capacity of the Diapak C18 (0.05–2.0 μg/L).

### 2.4. Separate Determination of Organic and Inorganic Forms of Tin in Water

**Figure 7:**
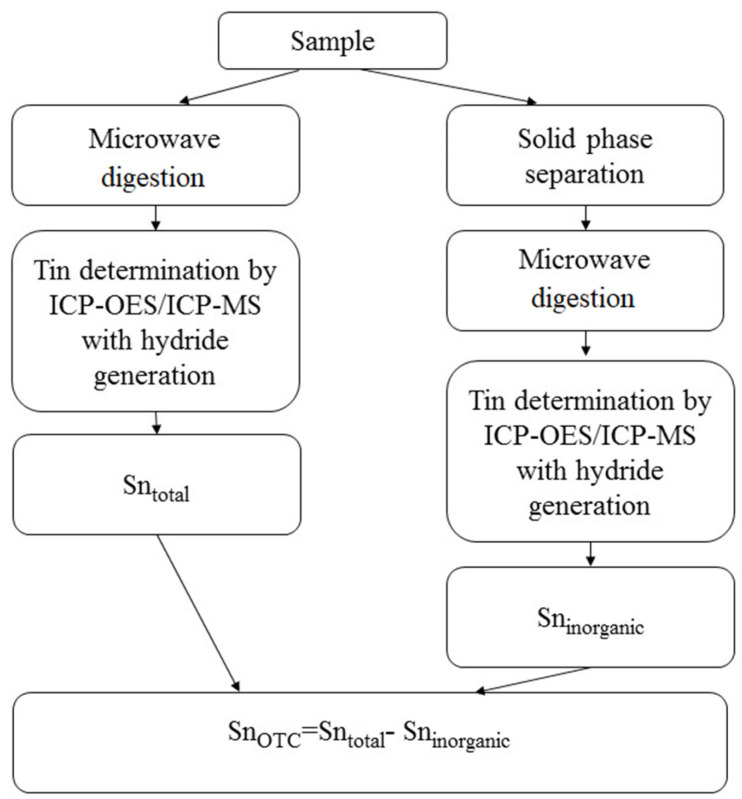
Scheme for the determination of tin forms by ICP spectrometry.

Considering the obtained results of optimization of the schemes for determining the total tin content in surface waters of different salinities, a scheme for the separate determination of organic and inorganic forms of the analyte in their joint presence was proposed (Figure 7). To do this, the total tin content in water samples was determined with ICP spectrometry with hydride generation after microwave mineralization of thermostable OTCs and conversion into inorganic tin. ICP-spectrometric determination of the inorganic form of the analyte in natural water samples was carried out after preliminary separation of the organic and inorganic forms using the Diapak C18 sorbent at pH = 10 and subsequent microwave mineralization of the solution. The OTC content in the waters was determined through the difference between the total tin content and the concentration of its inorganic form.

The accuracy of the selective solid-phase separation of the chemical forms of tin in their joint presence in model water samples as well as the determination of the total content of OTCs was confirmed through the analysis of water samples with different salinities containing tin (IV) chloride and TBT, TeBT, TMT, and MPT (Table 4). The concentrations of analytes in the additives were the same and amounted to 0.25, 0.50, and 1.0 μg/L in terms of tin. The Diapak C18 under optimized conditions provided quantitative separation of the inorganic and organic forms of tin in their joint presence in water. The total tin content as well as the concentration of the inorganic form in the analyzed solutions were determined using ICP-MS and ICP-OES with hydride generation. The total content of OTCs in water was determined through the difference between the found values of total and inorganic tin. The lower limits of OTC quantification were 0.03 μg/L (ICP-MS) and 0.05 μg/L (ICP-OES).

## 3. Materials and Methods

### 3.1. Research Objects

The objects of the study were model samples of waters with component compositions and salinity levels close to the waters of the Azov [36] and Black [37] Seas. Model samples were prepared on deionized water using the reagent grades recommended in [38].

When studying the influence of the matrix on the determination of tin, we were guided by the fact that the chemical composition and ratios of the main macro components in seawaters in all regions of the globe are considered to be the same in accordance with the Marcet principle [39]. Model seawaters with the chemical composition, ratios of the main macro components, and salinities of the Azov and Black Seas were prepared considering the data of [36,37] to adequately assess their effect on the determination of the speciations of tin.

### 3.2. Reagents

For experimental studies, high-purity argon (99.998%) was used; 15.4 mol/L HNO_3_, 12 mol/L HCl, 10 mol/L H_2_O_2_, tributyltin chloride (TBT, 98%), trimethyltin chloride (TMT, 98%), monophenyltin trichloride (MPT, 98%), and tetrabutyltin (TeBT, 94%) were purchased from Sigma-Aldrich (St. Louis, MO, USA); sodium borohydride (NaBH_4_, 96%) was purchased from PanReac AppliChem (Chicago, IL, USA); sodium chloride (NaCl, 99.9%), magnesium chloride 6-water (MgCl_2_ × 6H_2_O, 98.0%), sodium sulfate anhydrous (Na_2_SO_4_, 99.5%), anhydrous calcium chloride (CaCl_2_, 96.5%), potassium chloride (KCl, 99.8%), sodium carbonate acid (NaHCO_3_, 99.8%), and boric acid (H_3_BO_3_, 99.9%) were used.

All solutions were prepared in deionized water with a maximum resistivity of 18.2 MΩ cm^−1^ obtained in a sub-boiling distillation unit DuoPUR Sub-boiling Distilling System (Milestone, Milan, Italy). Calibration solutions in the range of 0.05–20,000 μg/L for ICP-OES and 0.01–100 μg/L for ICP-MS were prepared using a stock tin solution (C_Sn_ = 1 g/L) purchased from Inorganic Ventures (Christiansburg, VA, USA) with 1% HCl.

The influence of the matrix effects and ways to eliminate them in the determination of tin were studied using stock solutions of sodium, potassium, calcium, magnesium, sulfate ions, nitrate ions, and phosphate ions with a concentration of 1 g/L and chloride ions with a concentration of 10 g/L purchased from Inorganic Ventures (USA). The model seawater solution consisted of 22.00 g of NaCl, 9.70 g of MgCl_2_ × 6H_2_O, 3.70 g of Na_2_SO_4_, 1.00 g of CaCl_2_, 0.65 g of KCl, 0.20 g of NaHCO_3_, and 0.023 g of H_3_BO_3_ dissolved in 1 L deionized water.

Determination of tin through hydride generation was carried out using 0.50 mol/L sodium borohydride, stabilized with a solution of 0.10 mol/L sodium hydroxide, which was prepared on the day of analysis by dissolving a portion of NaBH_4_ (m = 1.90 g) in 100 mL of deionized water. Solutions of the oxidizing agents 15.4 mol/L HNO_3_; 12 mol/L HCl; 17.9 mol/L H_2_SO_4_; and 26.4 mol/L formic (CH_2_O_2_), 17.4 mol/L acetic (C_2_H_4_O_2_), and tartaric acids (C_4_H_6_O_6_, 99.9%) were prepared by diluting the acids with deionized water to the required concentrations in the ranges of 0.05–1.00 mol/L for mineral and 0.05–5.00 mol/L for organic acids.

Modeling of the samples containing OTCs in deionized water was carried out considering their possible content at the level of maximum permissible concentrations in the water areas of the studied aquatic ecosystems.

### 3.3. Instrumentation

The measurements were performed with the inductively coupled plasma mass spectrometer iCAP RQ (Thermo Scientific, Waltham, MA, USA) and the inductively coupled plasma optical emission spectrometer iCAP-7400 series (Thermo Scientific, Waltham, MA, USA). The data were acquired and processed with the ICP-MS Qtegra software (version 2.8.3170.309, Thermo Fisher Scientific, Waltham, MA, USA) and ICP-OES Qtegra software (version 2.8.2944.202, Thermo Fisher Scientific). The operating conditions of the devices, considering the specifics of the analyzed object, were studied using a MicroMist concentric nebulizer purchased from Glass Expansion (Melbourne, Australia).

#### 3.3.1. Optimization of Operating Modes of Spectrometers

To study the analytical characteristics of the ICP-MS and ICP-OES determinations of tin in water, the operating parameters of the spectrometers (the rates of cooling, auxiliary and sample argon flows, and the power of the RF generators) were optimized to achieve the best sensitivity, reproducibility, and accuracy of analysis. The above parameters of instrument operation were optimized by analyzing solutions with a constant tin concentration prepared in deionized, model sea- and freshwaters. The optimal operating parameters of the spectrometers are summarized in Table 5.

#### 3.3.2. Conditions for the Generation of Tin Hydrides

The operation parameters of the spectrometers under the conditions of hydride generation were optimized to obtain the maximum ratio of the analytical signal of tin to the background signal (Table 6). Hydrides were generated with sodium borohydride (NaBH_4_) using hydrochloric acid as an oxidizing agent. Tin hydrides were prepared using a commercial Thermo Fisher Scientific (Waltham, MA, USA) advanced hydride system. In an acrylic reaction cell filled with glass beads to increase the reaction yield, tin hydrides were formed by mixing the reagents introduced into the hydride system in parallel with the sample solution acidified to pH = 2.0. The resulting volatile compounds were transported to the plasma torch of the spectrometers with an argon flow through a membrane filter with a Teflon surface, which served as a gas–liquid separator.

#### 3.3.3. Calculation of the Limit of Quantification of Tin

The limits of quantification of inorganic tin (LOQ) were determined using the calibration graphs based on reference solutions prepared in deionized water and model seawaters of different salinities. For measuring the mean level and standard deviation of blank values, repeated measurements (n = 15) of blank solutions with corresponding salinities were carried out. The LOQ was obtained using solutions containing tin (IV) chloride. The tin LOQ was calculated as [40]:

LOQ = 10 × S/b,(1)

where S is the standard deviation of blank values at a confidence interval *p* = 0.95 and b is the tangent of the slope of the calibration graphs.

#### 3.3.4. Separation of Inorganic and Organic Tin in Water

To determine the inorganic and organic forms of tin in natural waters, the conditions for their separation were studied. Organotin compounds can be separated from inorganic tin by changing the chloride counterions of the OTCs to fluorides. In this case, low-dissociation complex compounds of the OTCs are formed [41,42], while inorganic tin (IV) halides remain in the water [14]. These properties of OTCs were considered when they were precipitated from aqueous solutions with potassium fluoride or iodide. The conversion of OTC fluorides into a low-dissociation form was evaluated using model solutions of deionized water with tin (IV) chloride; a mixture of TBT, TeBT, TMT, and MPT; and a mixture of OTCs with tin (IV) chloride with a total tin addition of 1.0 μg/L. The concentration range of potassium fluoride or iodide was chosen from 1.0 μg/L to 4.0 g/L in terms of halides, considering the stoichiometry of complex formation under conditions of excess halide.

Aqueous solutions of ammonia and iron (III) chloride can precipitate inorganic tin (IV) compounds while maintaining water-soluble forms of OTCs. Inorganic tin forms sparingly soluble compounds—hydrates of tin (IV) dioxide in two main forms: *α*-oxides (*α*-tin acid) and *β*-oxides (*β*-tin acid). These compounds are water-insoluble amorphous precipitates with products solubility of ~8 × 10^−33^ [14]. To test this approach, the inorganic speciation of tin was precipitated with aqueous solutions of ammonia and iron (III) chloride in the form of tin oxide [15].

The liquid–liquid separation of inorganic and organic forms of tin was studied by isolating OTCs from the aqueous phase with organic solvents often used for this purpose: dichloromethane and hexane [12].

The solid-phase separation of speciations of tin was carried out using concentrating cartridges based on polymer and silica gel sorbents with different functional groups:

i. Hydrophobic silica gel sorbent with grafted octadecyl groups (40–63 μm, pore diameter of 6 nm, sorbent weight of 600 mg) Diapak C18 (Biohimmak, Russia);
ii. Polymeric sorbent with the reversed-phase retention mechanism with a hydrophilic-lipophilic balance (31.4 μm, pore diameter of 8.2 nm, sorbent weight of 100 mg) Waters Oasis HLB 1cc (Waters Corporation Milford, Milford, MA, USA);
iii. Silica gel sorbent with a grafted octadecyl group with the reversed-phase retention mechanism Strata C18-E (Phenomenex, Torrance, CA, USA), acting due to strong hydrophobic and insignificant secondary polar interaction of active silanol groups (55 µm, pore diameter of 7.0 nm, sorbent weight of 100 mg);
iv. Functionalized silicon dioxide based on the trifunctional silane Isolute C18 (EC) (BCM Diagnostics, San Diego, CA, USA), which provides the main non-polar retention of analytes and the secondary interaction of silanol groups (50 μm, pore diameter of 6.0 nm, sorbent mass of 100 mg);
v. Sorbent with mixed action Isolute HAX (BCM Diagnostics, San Diego, CA, USA)—non-polar interaction due to octyl group C8 and strong anion-exchange mechanism of retention by quaternary amine -NR^3+^ (50 µm, pore diameter of 6.0 nm, sorbent mass of 200 mg).

The sorbents were conditioned with deionized water and methanol, and the conditions for separating tin forms at different pH levels were controlled with hydrochloric acid and ammonia solutions.

## 4. Conclusions and Future Perspectives

The described investigations enabled the separate determination of inorganic and organic forms of tin and the total content of tin in waters with different salinities using ICP spectrometry with hydride generation. In this work, we studied various approaches to the separation of tin forms: liquid–liquid extraction with various solvents, precipitation with halides or aqueous solutions of ammonia and iron (III) chloride, as well as solid-phase separation. The separation of the tin forms with the liquid–liquid extraction of the OTCs turned out to be inefficient due to the incomplete extraction of analytes and the partial extraction (up to 15%) of the inorganic tin into the organic phase. The precipitation of low contents of inorganic and organic forms of tin with halides, ammonia, and iron (III) chloride also proved to be inefficient. Separation of the tin forms was achieved using the silica gel sorbent Diapak C18, which, under optimized conditions, selectively extracted more than 95% of the OTCs from waters with different salinities with a minimal co-extraction of inorganic tin.

Based on the results of the research, a method was developed for determining the chemical forms of tin in natural waters after their solid-phase separation using the combined methods of ICP spectrometry with hydride generation and microwave digestion. The lower limits of quantification of the inorganic form of tin after its solid-phase separation from organotin compounds were 0.03 μg/L (ICP-MS) and 0.05 μg/L (ICP-OES). This makes it possible to separately determine inorganic and organic forms of tin in waters with different salinities. The total content of OTCs in water was determined through the difference between the values of total and inorganic tin. The technique will allow a comprehensive assessment of the toxicological impact of tin on the aquatic ecosystem. Considering the fact that the toxic properties of OTCs are still different, we can assume that the next phase in the development of these studies will be the separation and determination of tin compounds that are close in hazard class.

## Figures and Tables

**Table 1:** Guiding water quality standards and regulatory limits for tributyltin in marine water.

Organizations Regulating the Use of OTCs and the Name of the Document	Tributyltin Content	Source
European Community//Directive 2008/105/EC of the European Parliament and of the Council of 16 December 2008	0.2 ng/L annual average and 1.5 ng/L maximum allowable concentration	[14]
OSPAR Convention for the protection of the marine environment of the North East Atlantic OSPAR/ICES Workshop on the evaluation and update of background reference concentrations (BRCS) and ecotoxicological assessment criteria (EACS) and how these assessment tools should be used in assessing contaminants in water, sediment, and biota (Update 2004)	0.1 ng/L water concentration	[15]
United States Environmental Protection Agency. Ambient Aquatic Life. Water Quality Criteria for Tributyltin	One-hour average concentration not exceeding 420 ng/L more than once every three years (acute criterion). Four-day average concentration does not exceed 7.4 ng/L more than once every three years (chronic criterion)	[16]
Australia. Toxicant Guidelines for the Protection of Aquaculture Species (under review). Australian Sediment Quality Guidelines for TBT	Saltwater production: <10 ng/L	[17]
Canadian water quality guidelines for the protection of aquatic life//Organotins: tributyltin, triphenyltin, and tricyclohexyltin, 1999	1 ng/L	[18]
IMO’s Marine Environment Protection Committee (MEPC)	Prohibition of application	[13]

**Table 2:** Determination of tin forms by precipitation (n = 3, *p* = 0.95).

Deposition Method	Total Tin Concentration in Tested Samples/Mixtures, µg/L	*C_halide ion_*, g/L	Found Analyte Concentration, µg/L	
			ICP-OES	ICP-MS
OTC precipitation with fluorides	Mixture of OTC—1.0 µg/L	0.5	0.9 ± 0.2	1.0 ± 0.2
		1.0	0.9 ± 0.2	0.9 ± 0.2
		2.0	1.0 ± 0.2	0.9 ± 0.2
		3.0	0.9 ± 0.2	0.9 ± 0.2
	Mixture of tin (IV) chloride and OTC—1.0 µg/L	0.5	0.9 ± 0.2	0.9 ± 0.2
		1.0	0.9 ± 0.2	0.9 ± 0.2
		2.0	0.9 ± 0.2	0.9 ± 0.2
		3.0	0.9 ± 0.2	0.9 ± 0.2
OTC precipitation with iodides	Mixture of OTC—1.0 µg/L	0.5	1.0 ± 0.2	0.9 ± 0.2
		1.0	0.9 ± 0.2	1.0 ± 0.2
		2.0	1.0 ± 0.2	0.9 ± 0.2
	Mixture of tin (IV) chloride and OTC—1.0 µg/L	0.5	0.9 ± 0.2	0.9 ± 0.2
		1.0	1.0 ± 0.2	1.0 ± 0.2
		2.0	0.9 ± 0.2	1.0 ± 0.2
Precipitation of Sn^4+^ aqueous ammonia solution and iron (III) chloride	Tin (IV) chloride—1.0 µg/L	–	0.9 ± 0.2	0.9 ± 0.2
	Mixture of tin (IV) chloride and OTC—1.0 µg/L	–	0.9 ± 0.2	0.9 ± 0.2

**Table 3:** The content of inorganic tin in solutions of deionized water after solid-phase separation of OTCs under conditions of their different ratios (n = 3, *p* = 0.95).

Added Inorganic Form of Tin, µg/L	Added OTCs in Terms of Tin, µg/L	Ratio of Inorganic Tin to OTCs	Found Tin Content, µg/L	
			ICP-OES	ICP-MS
0.05	0.20	1:4	0.04 ± 0.01	0.05 ± 0.01
0.05	0.05	1:1	0.05 ± 0.01	0.05 ± 0.01
0.10	0.05	2:1	0.10 ± 0.02	0.11 ± 0.02
0.25	0.05	5:1	0.24 ± 0.05	0.26 ± 0.05
0.50	0.05	10:1	0.51 ± 0.10	0.48 ± 0.10

**Table 4:** The content of inorganic tin in solutions of deionized water after the solid-phase separation of OTCs under conditions of their different ratios * (n = 3, *p* = 0.95).

Type of Water	Introduced in Terms of Tin, µg/L	Found with ICP-MS, µg/L			Found with ICP-OES, µg/L		
		C_Σ_	C_IT_	Calculated C_OTC_	C_Σ_	C_IT_	Calculated C_OTC_
Deionized water	0.25	0.25 ± 0.05	0.04 ± 0.01	0.21 ± 0.04	0.25 ± 0.05	0.05 ± 0.01	0.20 ± 0.04
	0.50	0.48 ± 0.10	0.09 ± 0.02	0.39 ± 0.08	0.51 ± 0.10	0.10 ± 0.02	0.41 ± 0.08
	1.00	1.01 ± 0.20	0.21 ± 0.04	0.80 ± 0.15	1.05 ± 0.20	0.22 ± 0.04	0.83 ± 0.15
Model seawater with salinity of 6 ‰	0.25	0.24 ± 0.05	0.05 ± 0.01	0.19 ± 0.04	0.26 ± 0.05	0.05 ± 0.01	0.21 ± 0.04
	0.50	0.51 ± 0.10	0.11 ± 0.02	0.40 ± 0.08	0.54 ± 0.10	0.11 ± 0.02	0.43 ± 0.08
	1.00	1.01 ± 0.20	0.19 ± 0.04	0.82 ± 0.15	0.99 ± 0.20	0.19 ± 0.04	0.80 ± 0.15
Model seawater with salinity of 18 ‰	0.25	0.26 ± 0.05	0.04 ± 0.01	0.22 ± 0.04	0.26 ± 0.05	0.06 ± 0.01	0.20 ± 0.04
	0.50	0.50 ± 0.10	0.10 ± 0.02	0.40 ± 0.08	0.49 ± 0.10	0.09 ± 0.02	0.40 ± 0.08
	1.00	0.99 ± 0.20	0.20 ± 0.04	0.79 ± 0.15	1.00 ± 0.20	0.21 ± 0.04	0.79 ± 0.15

C_Σ_—total tin content; C_IT_—inorganic tin content; C_OTC_—total OTC content. Notes: * ratios of OTCs, tin (IV) chloride in a deionized water solution containing tin (IV) chloride, TBT, TeBT, TMT, and MPT are equimolar.

**Table 5:** Optimal operating parameters of the spectrometers.

Parameter		iCAP RQ Mass Spectrometer	iCAP-7400 Spectrometer (Axial Overview of Plasma)
Analyte		^120^Sn	Sn II 189.989 nm
Applied power, W		1300	1150
Argon flow rate, L/min	Plasma-forming (cooling)	15	12
	Auxiliary	0.80	0.50
	Nebulizer	1.10	0.50
Peristaltic pump speed, rpm		40	50
Sample rate, mL/min		0.4	

**Table 6:** Optimal operating parameters for spectrometers under conditions of tin hydride generation.

Parameter		iCAP RQ Mass Spectrometer	iCAP-7400 Spectrometer (Axial Overview of Plasma)
Analyte		^120^Sn	Sn II 189.989 nm
Applied power, W		1300	1150
Argon flow rate, L/min	Plasma-forming (cooling)	15	12
	Auxiliary	0.80	0.50
	Nebulizer	0.45	
Peristaltic pump speed, rpm		60	30
Sample introduction		Hydride system: oxidizer—0.10 mol/L HCl; reducing agent—0.50 mol/L NaBH_4_	

## Data Availability

Data included in article/referenced in article.

## Sample Availability

Not available.
